# Negative emotional status and influencing factors among young employees in center of disease control and prevention

**DOI:** 10.1186/s12889-022-12806-9

**Published:** 2022-02-25

**Authors:** Lu Han, Qiyu Li, Yu Zhang, Tuo Liu, Ran Niu, Qi Wang, Lina Zhao

**Affiliations:** 1 Chinese Center for Disease Control and Prevention, Beijing, 102206 China; 2 Jinzhou Medical University, Jinzhou, 121000 China; 3 Gansu Center for Disease Control and Prevention, Lanzhou, 730000 China; 4 National Institute for Occupational Health and Poison Control, Chinese Center for Disease Control and Prevention, Beijing, 100050 China; 5 National Institute for Nutrition and Health, Chinese Center for Disease Control and Prevention, Beijing, 100050 China; 6 Xinjiang Center for Disease Control and Prevention, Urumqi, 830002 China

**Keywords:** Young employees, CDC, Negative emotions, Influencing factors

## Abstract

**Background:**

Negative emotions among employees have become a public problem that increase the risk of developing the disease and accelerate its progression. This study aimed to investigate the status and influencing factors of negative emotions among young employees in center of disease control and prevention.

**Methods:**

Participants included 6099 employees aged 40 or below in center of disease control and prevention (CDC) of 32 province of China were interviewed by online questionnaire survey. The emotional conditions of anxiety and depression, and their influencing factors were analyzed.

**Results:**

A total of 5353 valid questionnaires were collected with the recovery rate of 87.77%. 2871 cases of young employees had different degrees of negative emotions at work, accounting for about 53.60%. Regression analysis showed that gender, professional title, educational level, job satisfaction, chronic diseases, daily sleep duration, average weekly overtime, physical activity time, and sugary beverage intake were the influencing factors of negative emotions (*P* < 0.05). Male, primary and below, never working overtime and daily physical activity time more than 30 min were protective factors for negative emotions (OR vale were 0.79, 0.68, 0.39 and 0.63, respectively, *P* < 0.05). Bachelor degree or above, poor job satisfaction, chronic disease, daily sleep duration less than 8 h and drinking one to three sugary drinks a week were the risk factors for negative emotion (OR vale were1.21, 4.32, 2.16, 2.75 and 1.20, respectively, *P* < 0.05).

**Conclusion:**

Due to the influence of work pressure, lifestyle, chronic diseases and other factors, young employees in CDC have a certain degree of negative emotions at work, which should be paid enough attention. Meanwhile, corresponding measures should be taken according to the influencing factors to reduce the occurrence of negative emotions.

## Background

Negative emotions such as depression and anxiety caused by workplace stress have become important factors that increase the risk of developing the disease and accelerate its progression [1]. Previous studies have found that, medical and health practitioners are more prone to occupational stress and burnout because of high workload and strained interpersonal relationship [2]. It develops further when there is no relief, developing a tendency for negative emotions in the form of anxiety and depression at work [3]. The survey on perceived stress of residents in 15 provinces of China shows that the perceived stress of adult residents is related to age, marriage, working status, income and physical activity [4]. Although studies have found the young employees in public health and disease control institutions often suffer from great work pressure, especially in the process of dealing with public health emergencies [5, 6]. However, they mainly focus on status of job burnout and bad mood [7], and there are few studies on large sample sizes of influencing factors. However, it is urgent to analyze the factors that cause the increasing occupational pressure of young practitioners at present. Disease prevention and control practitioners face great work pressure in the process of epidemic treatment, which is also prone to lead to the emergence of bad emotions. Haiyan He, et al. [8]. found that anxiety and depression were evident among CDC personnel during COVID-19, and these people need precise psychological intervention and humanistic care in order to have the best mental state to deal with the epidemic. On the other hand, the mental health status and influencing factors of CDC workers before the outbreak of the epidemic are also very important to understand the psychological changes before and after the epidemic. However, what was the mental health of young CDC workers before the outbreak of COVID-19?

This study was conducted in 2019, just before the outbreak of COVID-19, the main objective was to understand the situation of negative emotions in the daily work among young employees in CDC, and to analyze the influencing factors of this situation. This data can help us develop targeted health education programs to reduce the risk of related diseases. At the same time, it can also provide a data basis for studying the psychological changes and intervention measures of young CDC workers before and after the outbreak of COVID-19.

## Materials and methods

### Subjects

Six thousand ninety-nine employees aged 20 to 40 years from 32 provincial CDC were recruited to participate in this survey. The survey period was from October to November 2019. The protocols used in this study were approved by the Ethical Committee of Chinese Center for Disease Control and Prevention.

### Questionnaire design and survey methods

The questionnaire was designed by the research team based on the survey needs and previous research, and contained 72 questions from four dimensions, including basic information, ideological status, emotion and health-related behavior [9, 10], and details have been descried in our previous study [11]. Negative emotions at work include depression, anxiety and irritability [12]. Sleep duration in this paper refers to the average time of sleep per day during a week, the overtime, physical activity time and sugary drink intake are the average of overtime hours, exercise hours sugary drink amount during the week. In the design of the questionnaire, all sensitive questions are dealt with fuzzily.

In this study, the cluster sampling method was adopted to carry out a self-made online questionnaire survey, and relevant data were collected online by “scanning two-dimensional code or logging in to the survey link”. Before the survey, the investigators were trained and instructed to fill in the form online. The questionnaire data will be cleaned and coded by special personnel, and the questionnaires with inconsistent, incomplete and abnormal information will be eliminated.

### Statistical analysis

The results are presented as the mean values ± standard deviation (SD), One-way ANOVA was used for comparisons between groups. The influencing factors were analyzed by Two-category Logistic multifactor analysis. All of the statistical analyses were performed using the Statistical Product and Service Solutions13.0 software, and significance was set to the α = 0.05 error rate.

## Results

### Participant characteristics

A total of 5353 valid questionnaires were included and used for statistical analysis, accounting for 87.77% of the total questionnaires. There are 1886 cases (35.23%) males and 3467 (64.77%) cases female. 1670 cases (31.20%) were aged between 18 and 30 years, and 3683 cases (68.80%) were aged between 31 and 40 years.

### Status distribution of negative emotions

Two thousand eight hundred seventy-one cases had different degrees of negative emotions at work, accounting for about 53.63%, including 1958 cases of anxiety (68.20%), 429 cases of depression (14.94%), 484 cases of irritability (16.86%), shown as Tables 1 and 2. The proportion of negative emotion was higher in group of 31 ~ 40 years old, female, married, post-graduate degree and intermediate professional title. As shown in Table 2, the distribution differences of different types of negative emotions among age, gender, marital status and education level are statistically significant (*P* < 0.05), while the differences among professional titles are not statistically significant (*P* > 0.05).

**Table 1:** Distribution of negative emotions in daily work

	I/A		N/A	
	n	Percentage(%)	n	Percentage(%)
**Age (years)**				
18 ~ 30	904	31.49	766	30.86
31 ~ 40	1967	68.51	1716	69.14
**Gender**				
Male	983	34.24	903	36.38
Female	1888	65.76	1569	63.22
**Marital status**				
Married	2076	72.31	1831	73.77
Single	743	25.88	605	24.38
Other situations	52	1.81	46	1.85
**Education level**				
Bachelor degree or above	1207	42.04	1876	75.58
Post-graduate degree	1664	57.96	606	24.42
**Professional title**				
Primary and below	1220	42.49	1143	46.05
Intermediate	1228	42.77	985	39.69

**Table 2:** Difference of negative emotion rate under different characteristics

	Anxiety		Depression		Irritability		*F* value	*P* value
	n	Percentage(%)	n	Percentage(%)	n	Percentage(%)		
**Age (years)**								
18 ~ 30	626	69.25	146	16.15	132	14.60	13.79	0.00
31 ~ 40	1332	67.72	283	14.39	352	17.90		
**Gender**								
Male	643	65.41	176	17.90	164	16.68	11.60	0.01
Female	1315	69.65	253	13.40	320	16.95		
**Marital status**								
Married	1440	69.36	265	12.76	371	17.87	43.81	0.00
Single	489	65.81	153	20.59	101	13.59		
Other situations	29	55.77	11	21.15	12	23.08		
**Education level**								
Bachelor degree or above	745	61.72	190	15.74	272	22.54	53.63	0.00
Post-graduate degree	1213	72.90	239	14.36	212	12.74		
**Professional title**								
Primary and below	814	66.72	196	16.07	210	17.21	8.93	0.18
Intermediate	831	67.67	182	14.82	215	17.51		
Senior	313	74.00	51	12.06	59	13.95		

### Analysis results of influencing factors

Regression analysis showed that gender, professional title, educational level, job satisfaction, chronic diseases, daily sleep duration, average weekly overtime, physical activity time, and sugary beverage intake were the influencing factors of negative emotions (*P* < 0.05). Male, primary and below, never working overtime and daily physical activity time more than 30 min were protective factors for negative emotions (OR vale were 0.79, 0.68, 0.39 and 0.63, respectively, *P* < 0.05). Bachelor degree or above, poor job satisfaction, chronic disease, daily sleep duration less than 8 h and drinking one to three sugary drinks a week were the risk factors for negative emotion (OR vale were1.21, 4.32, 2.16, 2.75 and 1.20, respectively, *P* < 0.05), as shown in Table 3.

**Table 3:** Multiple regression analysis of influencing factors of negative emotion

Variable	*R* value	*P* value	OR	95%CI	
				Lower limits	Upper
**Intercept**	0.96	0.03	2.62	1.09	6.25
**Age (years)**					
18 ~ 30	0.17	0.17	1.18	0.93	1.49
31 ~ 40	0	–	1	–	–
**Gender**					
Male	−0.23	0.01	0.79	0.67	0.94
Female	0	–	1	–	–
**Education level**					
Bachelor degree or above	0.19	0.03	1.21	1.01	1.45
Post-graduate degree	0	–	1	–	–
**Department**					
Technical section	0.25	0.18	1.28	0.89	1.85
Administrative section	0.15	0.45	1.16	0.78	1.73
Others	0	–	1	–	–
**Professional title**					
Primary and below	−0.39	0.01	0.68	0.51	0.91
Intermediate	0.18	0.19	1.20	0.92	1.57
Senior	0	–	1	–	–
**Marital status**					
Married	0.27	0.38	1.30	0.72	2.35
Single	0.18	0.56	1.20	0.65	2.23
Other situations	0	–	1	–	–
**Job satisfaction**					
Dissatisfaction	1.46	0.00	4.32	2.73	6.86
Ordinary	0.87	0.00	2.39	1.94	2.95
Satisfaction	0	–	1	–	–
**Chronic disease**					
I/A	0.77	0.00	2.16	1.72	2.70
N/A	0	–	1	–	–
**Sleep duration**					
≤ 6 h per day	1.01	0.00	2.75	1.86	4.06
7 h per day	0.55	0.00	1.74	1.21	2.49
≥ 8 h per day	0	–	1	–	–
**Average overtime**					
Never	−0.93	0.00	0.39	0.27	0.58
0 ~ 10 h per week	−0.24	0.21	0.78	0.53	1.15
>10 h per week	0	–	1	–	–
**Physical activity time**					
≤ 30 min per day	−0.24	0.06	0.79	0.63	0.98
>30 min per day	−0.46	0.00	0.63	0.51	0.79
Never	0	–	1	–	–
**Sugary drink intake**					
1 ~ 3 bottle per week	0.18	0.04	1.20	1.01	1.43
≥ 4 bottle per week	0.13	0.44	1.14	0.82	1.57
Never	0	–	1	–	–

## Discussion

Due to the nature of medical and health work, practitioners suffer from a high level of work stress and psychological stress, many studies have shown that long-term high-load work can easily cause negative emotions and increase the risk of depression and chronic diseases [7, 13, 14]. CDC is the primary agency for dealing with public health emergencies, especially SAS, avian influenza and COVID-19 [15], in which young people are the main force. Therefore, paying attention to the physical and mental health of young practitioners is not only of great significance to individuals, but also to the overall quality of disease control.

Previous studies have found that during the COVID-19 pandemic, the proportion of employees in CDC with anxiety was 33.87% and that of with depression was 38.88% [8]. In this study, we found that the proportion of anxiety and other negative emotions among young practitioners was 53.60%, slightly lower than the results above, suggesting that the high workload brought by the epidemic increased the occurrence of negative emotions. Our study also found that the female employees have a higher proportion of negative situations, reaching 68.51%, which is basically consistent with the result (63.0%) obtained by Qiu Qianwen et al. in 2020 [7]. Walter Wurm et al. [16] in 2016 also found this phenomenon and believed that compared with men, women’s physical and mental health were more easily affected by the environment, so they were more prone to negative emotions.

In a survey of 1344 employees from four coal mines in Xinjiang, Xian Tingyong et al. [17]^.^ found that weekly working hours, positions and duties were significant factors contributing to increased occupational stress among practitioners. Our study also found that those who often work overtime are more likely to have negative emotions than those who never work overtime. This may be related to the fact that overtime takes up more spare time and young people are unable to obtain psychological relaxation from leisure time [18]. At the same time, the study found that physical activity of 30 min or more per day was a protective factor against negative emotions compared with those who did not exercise and those who rarely exercised. Ioannis D. Morres et al. [19]^.^ observed 19 adult women with depression who experienced significant relief after 4 weeks of preferred intensity exercise rather than prescribed intensity exercise. A survey of 7200 Chinese adolescents aged 13–18 years from six regions of China also found that screen and exercise time are associated with psychological symptoms in Chinese adolescents [20].

On the other hand, this study found that poor job satisfaction and daily sleep duration less than 8 h were risk factors for negative emotions. People with poor job satisfaction were more likely to experience negative emotions than those with higher job satisfaction, which may be related to complaining more about their jobs. Previous studies have found that the higher the occupational self-concept and dedication, the lower the incidence of occupational burnout [7]. Because stress comes from work overload and the inability to juggle work and family, people with high job satisfaction are more likely to find a balance and put more energy into their work.

Studies have found an association between the quality and duration of sleep and depression [21]. Healthy China Initiative (2019–2030) calls for mental health promotion actions to slow the rise of insomnia, anxiety and depression, and advocate getting 7–8 h of sleep a day [4]. Dieter Riemann et al. systematically analyzed the correlation between sleep quality and depression, believing that the two affect each other. Chronic sleep deprivation and poor quality sleep can lead to symptoms of depression, which can further worsen sleep quality [22]. In this study, it was also found that people who slept more than 8 h had a lower proportion of negative emotions, suggesting that lack of sleep was a risk factor. However, lack of sleep among CDC employees was also associated with heavier workloads and frequent overtime. Therefore, to address these problems fundamentally, consideration should be given to reducing the workload of young practitioners.

## Conclusion

There may be some bias in this study due to the influence of sample size, which may affect the accuracy and credibility of the results. Despite some limitations, our findings still represent a significant step forward, especially for finding out the possible influencing factors such as high work pressure, insufficient sleep and exercise time, chronic disease. Under the combined action of these factors, the young staff of the disease prevention and control institutions had certain negative emotions before the outbreak of COVID-19.

## Data Availability

The datasets used and analysed during the current study are available from the corresponding author on reasonable request.
